# Clinical approach to a child with poikiloderma: A case report

**DOI:** 10.1002/ccr3.4977

**Published:** 2021-10-17

**Authors:** Samir Shrestha, Sudha Agrawal

**Affiliations:** ^1^ Department of Dermatology and Venereology B.P. Koirala Institute of Health Sciences Dharan Nepal

**Keywords:** autosomal recessive, helicase gene, poikiloderma, Rothmund‐Thomson syndrome

## Abstract

There are various causes of childhood poikiloderma. A proper history and clinical examination may help to get conclusion and narrow down the differentials for the causes of poikiloderma.

## Graphical abstract

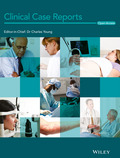

## 1 INTRODUCTION

Rothmund‐Thomson syndrome is a rare autosomal recessive condition presenting usually in infancy that can be diagnosed based on time of onset, spreading, and appearance of the poikiloderma. The purpose of reporting this case is to highlight the clinical approach to a child who presents with the features of poikiloderma. A proper history and clinical examination may help to get a conclusion among several rare cases of poikiloderma in a child. Rothmund‐Thomson Syndrome (RTS) is a rare autosomal recessive disorder, usually resulting from a mutation in the RecQL helicase gene that functions during DNA repair.^1^ It presents in infancy with erythematous macule or plaque often associated with blisters and edema that progress to develop poikiloderma initially involving the face and later to other sun‐exposed sites. The characteristic onset and progression of poikiloderma help to differentiate it from other causes of early‐onset poikiloderma.^2^ Furthermore, it is characterized by heterogeneous features such as short stature, skeletal abnormalities, cataract, premature aging, abnormalities of hair, nail, and teeth.^2^ It is associated with increased risk of osteosarcoma in childhood, cutaneous epithelial neoplasm, and hematological malignancies in adults.^2^

## 2 CASE REPORT

**FIGURE 1:**
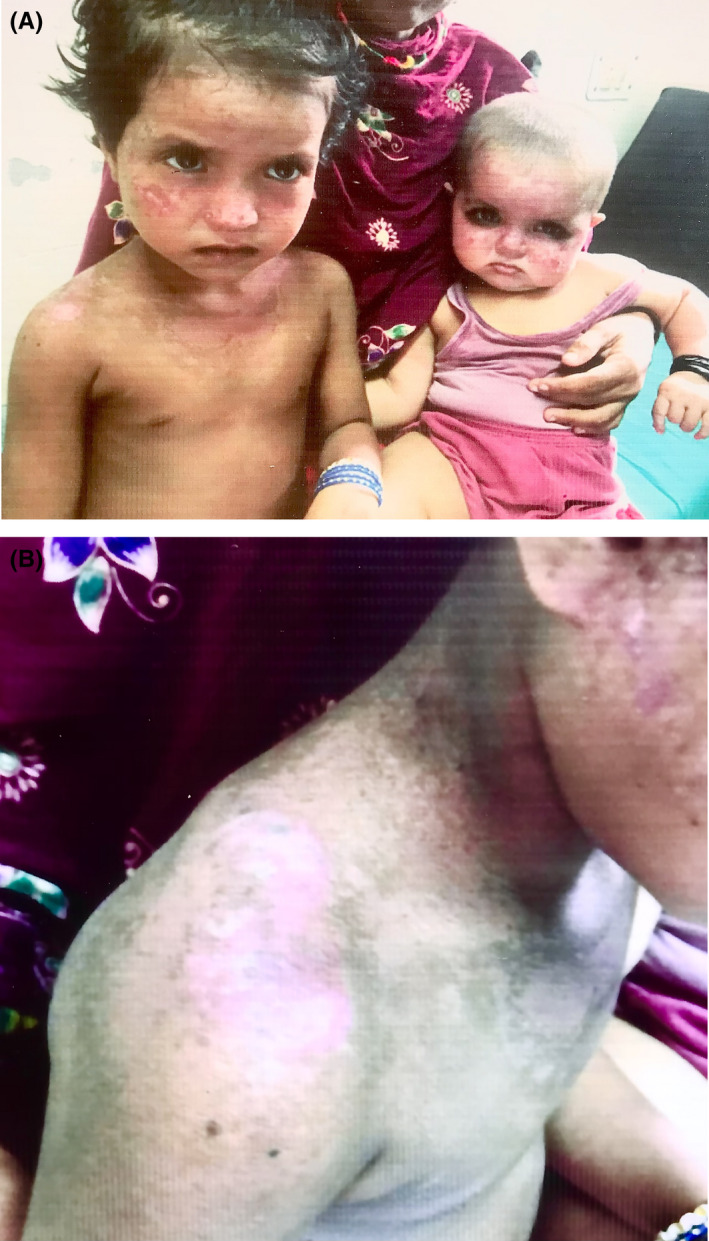
(A) showing erythematous plaques on her cheeks, bridge of nose, and V‐area of neck in both the siblings. (B) showing erythematous plaques along with atrophy, dyspigmentation, and telangiectasia on right shoulder

**FIGURE 2:**
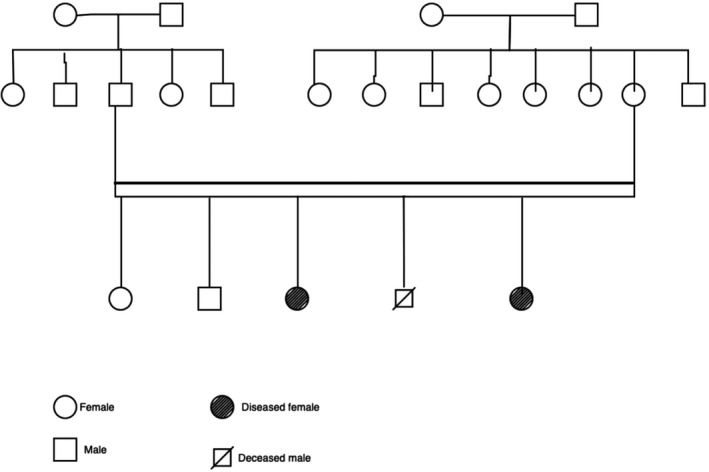
showing pedigree of the child

A 4‐year‐old girl presented with a rash on her face that was noticed by her mother 1 month following birth. She was 3^rd^ of the 5 children born at full term via normal vaginal delivery at home to consanguineous parents. Initially, the mother noticed erythematous macules and plaques on her cheeks that gradually developed areas of hypopigmentation and telangiectasia within 6 months (Figure 1A). Lesions with similar morphology and progression appeared to involve the shoulder (Figure 1B) followed by the V‐area of neck after a year that was aggravated on exposure to sunlight. There was no history of hair loss, recurrent respiratory infection, oral ulcer, feeding problems, dental, ocular, or neurological symptoms. On family history (as presented by pedigree in Figure 2), there was the demise of a 4^th^ child at birth and a history of a similar skin lesions on 8 months old 5^th^ child who developed the lesions at 2 months following birth (Figure 1A).

On cutaneous examination, there were multiple ill‐defined erythematous plaques on her cheeks, dorsum of the nose, V‐area of the neck and right shoulder with areas of dyspigmentation, atrophy, and telangiectasia. The height of the child was less than the 3^rd^ percentile for age and weight was at the 10^th^ percentile for age (as per WHO growth chart). There were no abnormalities detected on systemic examination. Baseline complete blood count, anti‐nuclear antibody, Comprehensive metabolic panel, and X‐ray of bilateral hand was normal. Karyotype analysis and gene sequencing was not done due to unavailability. RTS was diagnosed, and the patient was advised for sun‐protective behaviors and annual evaluation for the eyes, skin, and bone.

## 3 DISCUSSION

Rothmund‐Thomson Syndrome presents at 3 to 6‐month age with erythematous macule or plaque over face, sometimes associated with blisters and edema which gradually progress to involve extremities and gluteal regions sparing trunk and abdomen.^1^ Gradually, poikiloderma develops over the initial lesion that persists throughout the life. In a few of the cases, there might be systemic involvement that includes dental abnormalities (microdontia, conical teeth, frequent caries, and loss of teeth), ocular involvement (bilateral subscapular cataract), and bone abnormalities (absent or deformed radii, deformed ulnar bone, short hands and feet, aplasia of thumb, delayed bone age, and osteoporosis).^1^ There is an increased risk of osteosarcoma in childhood and epithelial neoplasms in adults.^2^ Patients have a normal life span unless associated with malignancy. Strict photoprotection and routine screening for malignancy are the mainstays of management.

Patients in our case had a characteristic rash of RTS developing at 1 month age, initially acute features of rash which developed into poikiloderma that was persistent with typical distribution pattern and had delayed growth. However, other systemic features of RTS were absent.

Detailed history and careful examination may aid in differentiating other causes of childhood poikiloderma. Acrogeria manifests at the time of birth or shortly afterward with poikiloderma limited to acral parts.^3^ Hereditary sclerosing poikiloderma presents in childhood with generalized poikiloderma with accentuation in flexures and extensor surfaces along with sclerodermatous plaques on palms and soles.^4^ Dyskeratosis congenita is characterized by the triad of severe nail involvement, poikiloderma conspicuous over the neck, and leucoplakia at a later age.^5^ Kindler syndrome is characterized by poikiloderma that develops at the age of 2 to 3 years in photo‐exposed sites along with acral blisters and mucosal stenosis.^2^ Patients with Cockayne's syndrome develop poikiloderma that spreads centripetally starting from distal limbs along with typical facies, limb abnormalities, wasting, and neurological manifestations.^6^ Rare disorders such as Bloom's syndrome, Fanconi's anemia, and Ataxia telangiectasia present with prominent telangiectasia and characteristic facies.^2^

## CONFLICT OF INTEREST

The authors have no conflicts of interest.

## AUTHOR CONTRIBUTIONS

SS involved in manuscript preparation and literature search and served as a correspondence author. SA involved in concept, manuscript editing, guidance, and final approval.

## CONSENT

A written consent was obtained from the parents of the patient for the publication of the case and images.

## Data Availability

We agree to make the manuscript available to general people and are also ready to provide other necessary data regarding the manuscript in case required.
